# Survey of Addiction Specialists’ Use of Medications to Treat Alcohol Use Disorder

**DOI:** 10.3389/fpsyt.2020.00047

**Published:** 2020-02-14

**Authors:** Jarrod Ehrie, Emily E. Hartwell, Paige E. Morris, Tami L. Mark, Henry R. Kranzler

**Affiliations:** ^1^ Department of Psychiatry, University of Pennsylvania Perelman School of Medicine, Philadelphia, PA, United States; ^2^ Mental Illness Research, Education, and Clinical Center of the Veterans Integrated Service Network 4, Crescenz Veterans Affairs Medical Center, Philadelphia, PA, United States; ^3^ Division of Behavioral Health and Criminal Justice Research, RTI International, Rockville, MD, United States

**Keywords:** alcohol use disorder, medication assisted treatment, survey, pharmacotherapy, addiction medicine, addiction psychiatry

## Abstract

**Objectives:**

Several medications have been shown to be safe and effective for treating alcohol use disorder (AUD); however, these medications are prescribed infrequently. We conducted a survey of the demographics, practice characteristics, and self-perceived knowledge, experience, and opinions of addiction specialists on the use of AUD medications and how to increase their use.

**Methods:**

We sent a 19-question survey to members of the American Society of Addiction Medicine (ASAM) and the American Academy of Addiction Psychiatry (AAAP).

**Results:**

We received a total of 395 responses from ASAM members and 194 responses from AAAP members. One hundred of the respondents were members of both organizations. The large majority of respondents (92.6%) were prescribers, and 81.6% were non-trainee physicians. The two most frequently used medications for treating AUD were oral naltrexone (27%) and long-acting naltrexone (18%). Respondents were significantly more confident in the strength of the research findings and evidence for the efficacy and safety of naltrexone than other AUD medications (p < 0.001 ). Respondents identified additional education to current providers about existing medications as the most important potential intervention to increase the use of AUD medications.

**Conclusions:**

Compared with a survey published in 2001, in 2018 the proportion of respondents who reported using naltrexone more than doubled and addiction specialists were more confident in their use of AUD medications, rating their efficacy and safety more highly. Consistent with findings from other recent studies, providing more education to practitioners about existing AUD medications may be the most effective way to increase their use.

## Introduction

Alcohol use disorder (AUD) is highly prevalent, with the past-year and lifetime prevalence in the adult U.S. population estimated to be 13.9 and 29.1%, respectively (1). AUD is associated with a variety of medical and psychiatric disorders, resulting in substantial disability (1) and increased mortality risk. In a large Swedish cohort study, the age-adjusted risk of mortality for patients with a diagnosis of AUD was 5.83 (2) and in the United States, alcohol consumption is associated with 88,000 deaths annually (3). Despite the high prevalence and adverse consequences of AUD, less than 20% of people diagnosed with the disorder in the United States ever receive treatment for it, with only 7.7% of affected individuals seeking treatment annually and 3.6% receiving care from a health care provider (1).

Three medications are approved by the Food and Drug Administration (FDA) for treating AUD: disulfiram, naltrexone (both oral and long-acting injectable formulations), and acamprosate. There is substantial evidence demonstrating the efficacy and safety of these medications in reducing alcohol consumption (4–8). Further, several medications (e.g., topiramate, gabapentin, and baclofen) approved in the United States for other indications have been shown to be efficacious in treating AUD (8). In 2015, the Substance Abuse and Mental Health Services Administration (SAMHSA) recommended that practitioners consider prescribing medication-assisted treatment in conjunction with a non-pharmacologic intervention to every patient with an AUD (9).

Despite the demonstrated benefits and the endorsement by multiple professional bodies of treating AUD pharmacologically (9–12), medications are not regularly prescribed for the disorder. Between 2002 and 2007, less than 9% of patients with AUD were prescribed an FDA-approved medication for the disorder (13), and from 2008 to 2009, only 3.4% of Veterans Health Administration patients diagnosed with AUD were prescribed one of these medications (14).

In 2001, Mark et al. (15) published the results of a survey of the memberships of the American Society of Addiction Medicine (ASAM) and the American Academy of Addiction Psychiatry (AAAP), which ascertained their knowledge, attitudes, and use of medications to treat AUD and elicited their opinions regarding barriers to the use of these medications (15). Greater than 70% of the physician respondents believed that they had a good-to-excellent ability to summarize the clinical research findings for disulfiram and oral naltrexone, the two drugs that were FDA-approved for treating AUD at that time. Greater than 60% of respondents reported feeling confident in their knowledge of the medications, with a majority reporting that the drugs' safety was good to excellent and that the research evidence favored their use. However, they reported prescribing naltrexone and disulfiram to only 13 and 9% of their AUD patients, respectively (15). When asked to identify the single best action to increase the use of AUD medications, 32.6% of respondents endorsed research to develop new medications, 16.8% endorsed educating providers about existing medications, 16.6% endorsed greater involvement of physicians in AUD treatment, and 4.6% endorsed making medications more affordable (15).

Since that survey was conducted, progress has been made in many areas. The FDA has approved two new pharmacotherapies for AUD: acamprosate and long-acting injectable naltrexone (5, 6). Meta-analyses of several other medications, including topiramate and baclofen, have shown them to be efficacious in treating AUD (8). Additional trials that evaluated existing medications provided further evidence of their safety and efficacy in treating AUD, and review articles and treatment guidelines called for increased prescribing of medications to treat the disorder (4, 7–12). Despite these advances, however, medications with demonstrated efficacy in treating AUD are still prescribed infrequently (14, 16).

To investigate the current prescribing habits of addiction specialists in treating AUD, we surveyed the memberships of ASAM and AAAP, modeling our survey on the earlier one by Mark et al. (15) to allow a direct comparison with it. We updated the instrument to include the medications approved for treating AUD over the past two decades. Our goal was to determine how self-reported prescription rates and opinions on the use of these medications have changed and why prescribers choose not to use medications to treat AUD patients. As a secondary aim, we sought to ascertain providers' experience and comfort with using genetic testing in their clinical practice, as pharmacogenetics is likely to play an important future role in treating AUD (17).

## Materials and Methods

### Respondents

All members of ASAM and AAAP who were registered to receive communications from either society were sent an email from the society with an embedded link to a Qualtrics survey. Respondents were informed that the purpose of the survey was to evaluate addiction specialists' opinions about and experience with medications used to treat AUD.

From mid-November through December 2018, members of ASAM were emailed a link to the survey a total of three times and members of AAAP were emailed a link to the survey twice (AAAP limits repeat emails to its membership). The interval between emails was 2 weeks. Individuals belonging to both organizations received an email from both but were asked to complete the survey only once. To reduce duplicate responses, the Qualtrics survey software prevented respondents from completing the survey twice using the same electronic device.

A total of 4,298 members of ASAM and 1,766 members of AAAP were invited to participate. A total of 489 individuals completed at least 30% of the survey (the criterion that we applied in deciding whether to report their responses). This included 395 ASAM members and 194 AAAP members, with 100 respondents belonging to both organizations, which represents an overall response rate of 8.4%.

### Survey Instrument

The online survey consisted of 19 questions and was designed to take approximately 10 minutes to complete. A copy of the survey is included in **Supplementary Data Sheet 1**. Demographic and professional information elicited in the survey included professional society membership (ASAM, AAAP, or both), provider type and practice level, board certification, percentage of professional time spent treating patients, number of patients treated, and the percentage of the practitioner’s patients treated for AUD.

Respondents were asked to choose an intervention that would be most effective in increasing prescribing of medications to treat AUD and to rate their confidence in their knowledge of the indications, contraindications, and adverse effects of the medications commonly used to treat AUD, their ability to summarize the clinical research findings for each medication, and how frequently they prescribed each medication to treat AUD. Respondents were queried about the primary reason that they did not prescribe a medication to all of their patients diagnosed with AUD. Respondents were then asked to rate the strength of the research evidence for each medication and the extent to which different patient characteristics affected their likelihood of prescribing an AUD medication to a patient.

Respondents also rated the effectiveness and the severity of side effects of each medication and to state their first and second choices of medication for promoting abstinence and for reducing alcohol consumption. In the last section of the survey, respondents were asked to rate how comfortable they were in ordering pharmacogenetic tests, whether they have access to genetics expertise for consultation, and whether they believed that their training adequately prepared them to order genetic tests and apply their results clinically. These questions were not specific to the use of AUD medications. Questions about the respondents' opinions and familiarity with medications for treating depression and opioid dependence were distributed throughout the survey to allow comparison with the AUD medications.

Because the respondents completed the survey anonymously, the University of Pennsylvania's Institutional Review Board determined that its content and administration were exempt from human subjects review.

### Incentive to Complete the Survey

To encourage respondents to complete the survey, they were informed that $5 would be donated to their professional society (ASAM or AAAP) for their completed survey, up to a maximum of $10,000.

### Statistical Analysis

Respondents who answered less than 30% (n = 30) of the survey were excluded. Those who identified themselves as physicians, nurse practitioners, physician assistants, residents, or fellows were considered to be prescribers (n = 464). We present only prescribers' responses for survey questions that pertain to prescribing medications or are specifically relevant for prescribers, as noted in the text or tables.

Participants who stated that they were board certified in psychiatry or in addiction psychiatry by the American Board of Psychiatry and Neurology (ABPN) were classified as psychiatrists. Answers that did not conform to the instructions listed in the survey questions were excluded.

We used t-tests and χ-squared tests to examine differences between groups or among response options. To reduce the risk of type 1 error due to multiple comparisons, the significance level was set at p < 0.001. Notation in the tables indicate the statistical methods used and the participants included in each analysis.

## Results

### Respondents’ Characteristics

The majority of respondents were non-trainee physicians (81.6%) and prescribers (92.6%) (**Supplementary Table 1**). The largest percentage of board certifications were for addiction medicine (29.1%) or addiction psychiatry (17.8%), which was expected given the organizations surveyed (**Supplementary Table 2**). On average, respondents spent the majority of their professional time treating patients [68.7% (SD = 31.6%)], followed by administration [16.7% (SD = 21.1%)], other activities [7.9% (SD = 16.4%)], and research [6.7% (SD = 16.0%)]. They reported treating an average of 168 unique patients in the previous 3 months, though this varied considerably (SD = 242). Of the patients treated, 26.2% (SD = 24.3%) were treated for AUD.

### Use of Medications

**Table 1** presents the average percentage of patients with AUD that the respondents treated with each medication. There were no statistically significant differences between psychiatrists and non-psychiatrists in the prescription rates for these medications.

**Table 1:** Frequency with which respondents prescribed medications to treat alcohol use disorder (AUD) (n = 344)^a^.

	All prescribers	Psychiatrists	Non-psychiatrist prescribers
Oral naltrexone	26.8	29.0	25.5
Long-acting naltrexone	17.8	14.0	20.0
Disulfiram	6.1	6.6	5.9
Acamprosate	7.6	7.0	8.0
Topiramate	4.2	5.2	3.7
Baclofen	1.5	0.9	1.9
Other^b^	14.6	14.6	14.2
None of the above	6.8	6.2	7.2

a Percentage of patients with AUD who were prescribed the listed medications. Only responses from prescribers were included in this analysis. Twenty-six percent of respondents did not complete this question. There was no statistically significant difference between psychiatrists and non-psychiatrists in the prescription rates for these medications.

b More than half of “other” responses included gabapentin. None of the differences shown in the table are statistically significant (i.e., p’s > 0.05).

### Respondents’ Knowledge of Medications Used to Treat Alcohol Use Disorder, Depression, and Opioid Use Disorder

As shown in **Table 2**, of all AUD medications, respondents were most confident in their knowledge of oral naltrexone, long-acting naltrexone, and disulfiram. Respondents had significantly more confidence in prescribing oral naltrexone than any other AUD medications. Notably, however, respondents were more confident in their knowledge of buprenorphine [for treating opioid use disorder (OUD)] than in their knowledge of any AUD medication.

**Table 2:** Prescribers’ ratings of confidence in their knowledge of medication’s indications, contraindications, and most frequent adverse events (n = 397)^a^.

	Never heard of medication	Not confident, but familiar with medication	Somewhat confident	Confident
Oral naltrexone for AUD	0	3.5	11.0	85.5
Long-acting naltrexone for AUD	0.3	7.0	14.8	77.9^b^
Disulfiram for AUD	0	10.8	17.9	71.3^b^
Acamprosate for AUD	1.5	10.4	19.9	68.2^b^
Topiramate for AUD	0.5	18.3	33.3	47.9^b^
Baclofen for AUD	2.8	38.9	28.9	29.4^b^
Gabapentin for AUD	0	15.8	22.6	61.6^b^
SSRI or SNRI for major depression	0	6.5	11.6	81.9^b^
Benzodiazepines for an anxiety disorder	0.3	9.6	14.4	75.7^b^
Buprenorphine for OUD	0.3	2.3	8.3	89.1^c^

a Percentage of respondents who endorsed each of the four possible responses; 14.5% did not complete this question.

b Based on a χ-squared test, the percent of respondents confident in the use of oral naltrexone for AUD was significantly greater (p < 0.001). than for each of the other medications except buprenorphine for opioid use disorder (OUD).

c Based on a χ-squared test, the percent of respondents confident in the use of buprenorphine for OUD was significantly greater (p < 0.001) than for oral naltrexone for alcohol use disorder (AUD).

The majority of respondents rated their ability to summarize the clinical research findings for oral naltrexone, long-acting naltrexone, disulfiram, and acamprosate as good to excellent (**Supplementary Table 3**). Respondents were less familiar with the clinical research findings for topiramate and baclofen. All of these differences were statistically significant. Respondents were also significantly more likely to rate their ability to summarize clinical research findings as excellent for selective serotonin reuptake inhibitors (SSRIs) and serotonin-norepinephrine reuptake inhibitors (SNRIs) for treating major depression [χ^2^(1) = 70.4, p < 0.001] and for buprenorphine for treating OUD [χ^2^(1) = 50.7, p < 0.001] than for any of the AUD medications.

### Respondents’ Opinions About Research Evidence on the Efficacy of Alcohol Use Disorder Medications

Respondents reported that there was strong research evidence supporting the use of oral and long-acting naltrexone but rated the research evidence for the other AUD medications less highly (**Supplementary Table 4**). A majority of respondents reported that there was evidence in favor of using all of the medications except baclofen for treating AUD. Respondents were more likely to state that long-acting naltrexone had strong evidence in favor of its use relative to oral naltrexone [χ^2^(1) = 136.9, p < 0.001] but were less likely to state that acamprosate [χ^2^(1) = 27.6, p < 0.001] or topiramate [χ^2^(1) = 11.2, p < 0.001] had strong evidence in favor of their use relative to oral naltrexone.

### Prescribers’ Opinions About the Efficacy and Safety of Alcohol Use Disorder Medications

As shown in **Table 3**, prescribers had a favorable view of the efficacy of naltrexone (both oral and long-acting) for treating AUD, though they rated the other AUD medications as less efficacious, to a statistically significant degree for disulfiram [χ^2^(1) = 13.2, p < 0.001] and acamprosate [χ^2^(1) = 54.7, p < 0.001]. A substantial proportion of prescribers said that they did not know how efficacious topiramate or baclofen were (17 and 31.5%, respectively). Disulfiram (17.0%) and baclofen (16.3%) were the medications most frequently rated as having poor efficacy. In contrast, 93.9% of respondents considered buprenorphine to be efficacious in treating OUD, with 64.6% of prescribers rating its efficacy as excellent, a statistically significant greater proportion than rated oral naltrexone excellent [χ^2^(1) = 18.7, p < 0.001].

**Table 3:** Prescribers’ ratings of the severity of side effects of medications for alcohol use disorder (AUD) and other psychiatric medications (n=354)^a^.

	Very severe/strongly limits prescribing	Severe/limits prescribing	Some/limits my prescribing	Few effects	None	Don't know
Oral naltrexone for AUD	0.6	1.4	23.6	69.1	2.8	2.5
Long-acting naltrexone for AUD	0.3	2.8	26.1	65.0	1.7	4.2^b^
Disulfiram for AUD	12.6	26.1	34.3	20.2	2.8	3.9^b^
Acamprosate for AUD	0.3	2.0	19.5	59.0	9.6	9.6^b^
Topiramate for AUD	0.6	9.3	48.0	27.1	1.4	13.6
Baclofen for AUD	1.4	5.4	31.4	27.7	2.8	31.4
SSRI or SNRI for major depression	0.3	0.8	29.7	60.5	5.1	3.7
Buprenorphine for opioid use disorder	0	1.1	14.9	70.2	11.8	2.0^b^

a Percentage of prescribers who endorsed each of the six possible responses. Twenty-four percent of prescribers did not complete this question.

b Based on a χ-squared test, the percentage of prescribers stating that the side effects of this medication limit their prescribing is significantly different from the corresponding percentage for oral naltrexone at P < 0.001.

The majority of prescribers indicated that adverse effects did not limit their prescribing of oral naltrexone, acamprosate, SSRIs, SNRIs, or buprenorphine (**Supplementary Table 5**). Prescribers were more likely to say that the side effects of long-acting naltrexone [χ^2^(1) = 119.2, p < 0.001] and SSRIs [χ^2^(1) = 11.5, p < 0.001] limited their prescribing relative to oral naltrexone, but prescribers felt the side effects of buprenorphine [χ^2^(1) = 11.3, p < 0.001] were less limiting than those of oral naltrexone.

### Patient Characteristics Influencing the Use of Medications

The three patient factors identified by more than 90% of respondents as most likely to increase their likelihood to prescribe an AUD medication were craving, the patient's willingness to comply with a medication regimen, and the patient’s request for an AUD medication (**Supplementary Table 6**).

### Reasons for Not Prescribing Alcohol Use Disorder Medications More Often

By far, the most common reason prescribers identified for not prescribing an AUD medication to patients diagnosed with AUD was the patients' refusal to comply with treatment (**Table 4**). Other reasons for not prescribing an AUD medication were its small effect on drinking relative to adverse effects, non-affordability, or substantial adverse effects.

**Table 4:** Most important reasons for not prescribing alcohol use disorder (AUD) medications to all patients with AUD diagnosis (n = 262)^a^.

Reason for not prescribing	Percentage selecting as most important
Patients refused to comply with treatment	56.4
Small effect on drinking relative to side effects	13.2
Patients could not afford medication	11.6
Concerns about side effects	10.0
Patients were not in a formal treatment program	7.2
Lack of time to prescribe and monitor prescriptions	1.6

a Average percentages for prescribers who saw patients with AUD in the last 3 months.

### Respondents’ Opinions About Barriers to Medication Use

The potential actions rated most important for promoting the use of medications to treat AUD were additional education to providers about existing medications, research to develop new medications, and greater involvement of physicians in AUD treatment (**Table 5**). Less than 10% of respondents thought that making medications more affordable, providing insurance coverage for medications, giving more education to patients about existing medications, or more research on existing medications were the most important actions to take.

**Table 5:** Most important actions that would results in wider use of medications to treat alcohol use disorder (AUD) (n = 412)^a^.

Potential action	Percentage selecting as most important
More education to providers about existing medications	33.0
More research to develop new medications	21.6
Increase involvement of physicians in AUD treatment	18.9
Make medications more affordable	9.5
Provide insurance coverage for medications	7.5
More education to patients about existing medications	7.3
More research on existing medications	2.2

a Sixteen percent of respondents did not respond to this question.

### Respondents’ Comfort and Familiarity With Pharmacogenetics

Less than one-third of respondents reported feeling comfortable ordering pharmacogenetic testing to predict the risk of adverse events or the likelihood of a treatment response (**Supplementary Table 7**). A similar proportion reported that they had access to genetics expertise when a question regarding pharmacogenetics arose, and less than one-fifth of respondents believed that their genetics training adequately prepared them to order testing and use the results clinically.

## Discussion

Respondents to this survey reported that more than one-quarter of their patients were being treated for AUD, of whom 27% were treated with oral naltrexone, more than double the 13% reported in 2001 (15). In contrast, only 6% of practitioners prescribed disulfiram, down from 9% in 2001 (15). The survey also showed that specialists' knowledge of and familiarity with AUD medications have increased substantially over time. This is most evident for oral naltrexone, where the proportion of respondents who reported feeling confident in its use increased from 66 to 86% between 2001 and 2018, the proportion who rated their ability to summarize the clinical research findings as “excellent” increased from 20 to 33%, and the percentage who rated the evidence as “strongly in favor of using” the medication increased from 40 to 68% (15). Respondents were more familiar and more confident in their use of naltrexone (both oral and long-acting) than in their use of other AUD medications.

The more familiar respondents were with a given medication, the more highly they rated its efficacy and safety and the more likely they were to prescribe it. One explanation for these findings is that information on more effective medications is more broadly disseminated and they are more widely available; an alternative explanation is that the more familiar respondents are with a medication and the data supporting its use, the more likely they are to rate it as effective and safe.

The majority of respondents identified patients' refusal to comply with treatment as the most important reason for not prescribing AUD medications. A meta-analysis of 771 intervention studies with adherence outcomes (18) showed that the most successful efforts to improve medication adherence are those that link taking the medication with existing daily routines and that use behavioral interventions, rather than cognitive strategies aimed at changing knowledge and beliefs. Effective behavioral interventions include prompts to take medications, self-administration practice, special packaging and labeling, behavioral contracts, and self-monitoring of adherence, all interventions that could readily be used to enhance adherence with AUD medications.

In addition, 23% of respondents pointed to limited efficacy or intolerable side effects as the most important factors limiting their prescribing of medications for AUD. Although these perceptions could reflect a lack of knowledge of the medications currently available, there is clearly a need to expand the available pharmacological armamentarium by adding novel agents and repurposing existing ones.

Although respondents opined that there was strong evidence in favor of using long-acting naltrexone and that treatment adherence was a key limiting factor in prescribing medications to treat AUD, they reported greater use of oral naltrexone than the long-acting formulation. Further, although respondents thought that there was stronger evidence in favor of using injectable naltrexone than oral naltrexone, the difference was small (73.2 *vs*. 67.8%, respectively). The explanation for these findings may lie in the high cost of long-acting naltrexone and the fact that some insurers require evidence of non-response to oral naltrexone before they will approve the use of the long-acting formulation for treating AUD.

Notably, respondents' confidence and familiarity in treating OUD with buprenorphine was significantly greater than for the use of any of the AUD medications for treating AUD. This may reflect the current opioid epidemic and the attendant widespread emphasis on medication-assisted treatment for OUD. The National Institute on Drug Abuse's Clinical Trials Network (CTN) has been a platform for demonstrating the clinical utility and practicability of buprenorphine (19) and from its initiation the CTN has recognized that best-practices research could facilitate the implementation of buprenorphine pharmacotherapy. A similar, community-focused effort to promote the use of pharmacotherapy for treating AUD is one potential approach to address the low rate of uptake of medication-assisted treatment of the disorder.

Nearly twice the percentage of respondents in the current survey as in the 2001 survey identified provider education about existing medications as most important for increasing AUD medication prescribing (33 *vs*. 17%). Although a lower priority, respondents in the current survey were also more likely than in the 2001 survey to identify making medications to treat AUD more affordable as a means to enhance prescribing (12 *vs*. 5%). In contrast, a smaller proportion of respondents in the current survey opined that more research to develop new medications was paramount (22 *vs*. 33% in 2001). This apparent shift in priority likely reflects the uptake of additional AUD medications during the past two decades (4), substantially augmenting the available therapeutic options, though in some cases, the new options (e.g., long-acting naltrexone) are substantially more costly. The findings from the current study are consistent with prior surveys of physicians (20) and focus groups of primary care providers (21), which identified a lack of familiarity with AUD medications as the main factor limiting the pharmacological treatment of AUD. Authors of those reports called for more training in the use of available medications as the highest priority for prescribers.

Recently a number of high-quality review articles and practice guidelines have been published that detail when and how to use medications in the treatment of AUD (4–12). An important question is how best to disseminate the findings to current and future prescribers. One possible approach is to develop condensed, user-friendly guides from existing literature and to disseminate them electronically to providers; an alternative would be to make more available continuing medical education (CME) courses that focus specifically on pharmacotherapy for AUD.

Survey responses also showed that addiction specialists are not comfortable ordering pharmacogenetic testing, do not believe that they have access to genetics expertise, and do not feel that they have adequate training in pharmacogenetics. Current research on treating AUD focuses on identifying genetic factors that could influence AUD treatment decisions (22), but those advances will need to be accompanied by efforts to increase providers' comfort with and knowledge of pharmacogenetic testing. CME courses on pharmacogenetics may be the most effective way to disseminate clinical genetics expertise rapidly and integrating principles of pharmacogenetics into training programs can help to prepare future generations of providers to handle this emerging approach to diagnosis and treatment.

The greatest limitation of the present study is the low response rate, which limits how representative the findings are of addiction specialists as a whole. The study by Mark et al., which provided a much larger incentive to respondents, had a response rate of 65% (15). Further, because only members of addiction-related societies were surveyed in this study, the results cannot be generalized to prescribers who are not addiction specialists. Because respondents were permitted to choose only one medication for medication-related questions in the survey, prescribers who frequently use a combination of medications to treat AUD were unable to indicate that preference.

The strengths of this study include its breadth (surveying many of the board-certified addiction specialists in the United States) and its depth (the survey included specific questions about respondents' knowledge, familiarity, comfort, and preferences for AUD medications). The survey was designed to query addiction specialists, similar to that by Mark et al. (15), whereas other recent studies have focused on the opinions and experiences of primary care physicians in treating AUD (20, 21). This allowed us to consider how the landscape of knowledge and attitudes regarding AUD treatment may have changed over the past two decades. As addiction specialists receive specialized training and are likely more experienced in managing AUD, their responses are less likely to be confounded by insufficient training and exposure to AUD treatment than in other practitioner groups. The view that additional training in the use of currently available medications is a key approach to enhancing prescribing of AUD medications is underscored by the fact that the respondents in this study were all addiction specialists.

In conclusion, perceptions of the pharmacological treatment of AUD appear to have changed over the past two decades. Addiction specialists report greater comfort and familiarity with the medications available for treating AUD and are prescribing the medications to their AUD patients much more frequently than was the case two decades ago. Nonetheless, there is a strong perceived need for additional training in the use of the available medications and concerns about their efficacy. Overall, the most feasible way to enhance the treatment of AUD may be to disseminate the results of high-quality clinical trials and meta-analyses of AUD medications to all clinicians who treat addiction and to incorporate this information into medical school and residency training curricula.

## Data Availability Statement

The dataset compiled for this study is available on reasonable request to the corresponding author.

## Ethics Statement

The studies involving human participants were reviewed and approved by the Institutional Review Board, University of Pennsylvania Perelman School of Medicine. The ethics committee waived the requirement of written informed consent for participation.

## Author Contributions

JE was responsible for drafting the grant application, IRB submission, and manuscript. JE, PM, and HK collaborated on the design of the survey and the specific questions: PM implemented the survey in the Qualtrics software and disseminated it to AAAP and ASAM members. EH conducted the statistical analysis of the resulting data and edited the manuscript. TM assisted with comparisons to the 2001 survey and edited the manuscript. HK conceived and supervised the project, including design of the survey and obtaining grant support for the project. He also reviewed and edited the grant application, IRB submission, and manuscript.

## Funding

The study was funded by NIAAA grant R01 AA023192 05S1 and by VISN 4 MIRECC of the Crescenz VAMC, Philadelphia, PA.

## Conflict of Interest

HK is a member of the American Society of Clinical Psychopharmacology's Alcohol Clinical Trials Initiative (ACTIVE Group), which during the past three years was supported by Abbvie, Ethypharm, Indivior, Lilly, Lundbeck, Mitsubishi, Otsuka, and Pfizer. HK is named as an inventor on PCT patent application #15/878,640 entitled: “Genotype-guided dosing of opioid agonists,” filed January 24, 2018.

The remaining authors declare that the research was conducted in the absence of any commercial or financial relationships that could be construed as a potential conflict of interest.
