# Supplementary material for: Survey of Addiction Specialists’ Use of Medications to Treat Alcohol Use Disorder
Source: Front Psychiatry. 2020 Feb 14;11:47. doi: 10.3389/fpsyt.2020.00047 (PMC7034336; doi:10.3389/fpsyt.2020.00047)
Supplement: Supplementary file 1 [file DataSheet_1.pdf]

A. PHYSICIAN CHARACTERISTICS

Thank you for taking the time to complete this survey. If you are a member of both the American Academy of Addiction Psychiatry and the American Society of Addiction Medicine, please take this survey only once.

A1. Are you a member of:

- ☐ American Academy of Addiction Psychiatry (AAAP)
- ☐ American Society of Addiction Medicine (ASAM)
- ☐ Both

A2. Are you a:

- ☐ Medical Student
- ☐ Resident or Fellow
- ☐ MD or DO (Non-trainee)
- ☐ NP or PA
- ☐ Psychologist
- ☐ Other

A3. Are you board certified in any specialty?

Choose all that apply

- ☐ NO, not board certified in any specialty
- ☐ Psychiatry (ABPN)
- ☐ Addiction Psychiatry (ABPN)
- ☐ Internal Medicine (ABIM)
- ☐ Family Practice (ABFM)
- ☐ Addiction Medicine (ABAM/ABPM)
- ☐ Other______________________________

A4. Do you consider yourself a specialist in treating addiction?

- ☐ Yes
- ☐ No

A5. Within the last 12 months, approximately what percentage of your employed time was spent treating patients, in administration, in research, or in other employed activities?

Treating patients	___ ___ ___ %
Administration	___ ___ ___ %
Research	___ ___ ___ %
Other activities	___ ___ ___ %
**TOTAL**	**1 0 0 %**

B. PATIENT CHARACTERISTICS

B1. Approximately how many unique patients have you treated in the last 3 months?

___ ___ ___ # of patients

B2. Approximately what percentage of your patients did you treat for alcohol use disorder (AUD) in the last 3 months? Please provide your best estimate.

____ ____ ___ % of patients

B3. Overall, approximately what percentage of all patients that you treat for AUD has the following types of insurance? Please provide your best estimate.

Private insurance	___ ___ ___ %
Medicaid	___ ___ ___ %
Medicare	___ ___ ___ %
Block Grant Coverage	___ ___ ___ %
Other ________________	___ ___ ___ %
No insurance	___ ___ ___ %
**TOTAL**	**1 0 0 %**

[Please check that percentages add to 100%]

C. OPINIONS ABOUT PHARMACOTHERAPIES FOR ALCOHOL USE DISORDER (AUD)

C1. In your opinion, what are the 3 most important actions that would result in wider use of pharmacotherapies to treat AUD?

Please rank your selections, where

1 = most important,

2 = next most important,

3 = next most important.

Please rank your selections (1, 2, 3)

___ More research to develop new medications

___ More research on existing medications

___ More education to providers about existing medications

___ More education to patients about existing medications

___ Make medications more affordable

___ Provide insurance coverage for medications

___ Increase involvement of physicians in AUD treatment

D. KNOWLEDGE AND USE OF MEDICATIONS

D1. For each of the following medications, please indicate how confident you are with your knowledge of the medication's indications, contraindications, and most frequent adverse effects.

	**Never heard of medication**	**Not confident, but familiar with medication**	**Somewhat confident**	**Confident**
**a. Oral Naltrexone (ReVia or Depade) for AUD**	☐	☐	☐	☐
**b. Long-acting Naltrexone (Vivitrol) for AUD**	☐	☐	☐	☐
**c. Disulfiram (Antabuse) for AUD**	☐	☐	☐	☐
**d. Acamprosate (Campral) for AUD**	☐	☐	☐	☐
**e. Topiramate (Topamax) for AUD**	☐	☐	☐	☐
**f. Baclofen (Lioresal) for AUD**	☐	☐	☐	☐
**g. Gabapentin (Neurontin) for AUD**	☐	☐	☐	☐
**h. SSRI (e.g., Zoloft) or SNRI (e.g., Effexor) for major depression**	☐	☐	☐	☐
**i. Benzodiazepines (e.g., Ativan) for an anxiety disorder**	☐	☐	☐	☐
**j. Buprenorphine (Suboxone or Sublocade) for opioid use disorder**	☐	☐	☐	☐

D2. Please consider each of the following medications. How would you characterize your ability to summarize the clinical research findings for each medication?

	**Never Heard of Medication**	**Poor**	**Fair**	**Good**	**Very Good**	**Excellent**
**a. Oral Naltrexone (ReVia or Depade) for AUD**	☐	☐	☐	☐	☐	☐
**b. Long-Acting Naltrexone (Vivitrol) for AUD**	☐	☐	☐	☐	☐	☐
**c. Disulfiram (Antabuse) for AUD**	☐	☐	☐	☐	☐	☐
**d. Acamprosate (Campral) for AUD**	☐	☐	☐	☐	☐	☐
**e. Topiramate (Topamax) for AUD**	☐	☐	☐	☐	☐	☐
**f. Baclofen (Lioresal) for AUD**	☐	☐	☐	☐	☐	☐
**g. SSRI or SNRI for major depression**	☐	☐	☐	☐	☐	☐
**h. Buprenorphine (Suboxone) for opioid use disorder**	☐	☐	☐	☐	☐	☐

D3. **Please consider each of the following medications. To approximately what percent of patients that you treated for AUD in the last 3 months did you prescribe each medication? Please provide your best estimate.** **If you did not prescribe any of the following medications in the last 3 months, please enter "100" next to the "None of the Above" option. THE TOTAL SHOULD NOT EXCEED 100%.**

**a. Oral Naltrexone (ReVia or Depade) for AUD**	___ ___ ___ %
**b. Long-Acting Naltrexone (Vivitrol) for AUD**	___ ___ ___ %
**c. Disulfiram (Antabuse) for AUD**	___ ___ ___ %
**d. Acamprosate (Campral) for AUD**	___ ___ ___ %
**e. Topiramate (Topamax) for AUD**	___ ___ ___ %
**f. Baclofen (Lioresal) for AUD**	___ ___ ___ %
**g. Other _____________**	___ ___ ___ %
**h. None of the above**	___ ___ ___ %
**Total:**	**TOTAL SHOULD NOT EXCEED 100%**

D4. **If you prescribed a medication to less than 100% of patients that you treated for AUD in the last 3 months, please rank the top 3 reasons why you did not prescribe it to some patients.**

Please rank your selections, where
1 = most important,
2 = next most important,
3 = next most important.

Please rank your selections (1, 2, 3)

_____ Small effect on drinking relative to side effects
_____ Concerns about side effects
_____ Patients could not afford medication
_____ Patients refused to take it or would not comply with treatment
_____ Patients were not in a formal treatment program
_____ Lack of time to prescribe and monitor prescriptions

D5. **How would you characterize the research evidence on the effects of the following medications to treat AUD?**

a. Naltrexone (ReVia)

☐ Strong evidence against using
☐ Weak evidence against using
☐ Mixed evidence
☐ Weak evidence in favor of using
☐ Strong evidence in favor of using
☐ Don't know

b. Long-Acting Naltrexone (Vivitrol)

☐ Strong evidence against using
☐ Weak evidence against using
☐ Mixed evidence
☐ Weak evidence in favor of using
☐ Strong evidence in favor of using
☐ Don't know

c. **Disulfiram (Antabuse)**

- ☐ Strong evidence against using
- ☐ Weak evidence against using
- ☐ Mixed evidence
- ☐ Weak evidence in favor of using
- ☐ Strong evidence in favor of using
- ☐ Don’t know

d. **Acamprosate (Campral)**

- ☐ Strong evidence against using
- ☐ Weak evidence against using
- ☐ Mixed evidence
- ☐ Weak evidence in favor of using
- ☐ Strong evidence in favor of using
- ☐ Don’t know

e. **Topiramate (Topamax)**

- ☐ Strong evidence against using
- ☐ Weak evidence against using
- ☐ Mixed evidence
- ☐ Weak evidence in favor of using
- ☐ Strong evidence in favor of using
- ☐ Don’t know

f. **Baclofen (Lioresal)**

- ☐ Strong evidence against using
- ☐ Weak evidence against using
- ☐ Mixed evidence
- ☐ Weak evidence in favor of using
- ☐ Strong evidence in favor of using
- ☐ Don’t know

g. **Gabapentin (Neurontin)**

- ☐ Strong evidence against using
- ☐ Weak evidence against using
- ☐ Mixed evidence
- ☐ Weak evidence in favor of using
- ☐ Strong evidence in favor of using
- ☐ Don’t know

D6. Please indicate the extent to which the following patient characteristics influence your decision to prescribe a medication to treat AUD.

	Effect Against Prescribing			Effect in Favor of Prescribing	
	Strong	Moderate	NO Effect	Moderate	Strong
a. Patient has experienced a previous relapse	☐	☐	☐	☐	☐
b. Patient is experiencing craving	☐	☐	☐	☐	☐
c. Patient has a comorbid mental illness	☐	☐	☐	☐	☐
d. Patient has impaired liver functioning	☐	☐	☐	☐	☐
e. Patient has a history of alcohol withdrawal	☐	☐	☐	☐	☐
f. Patient has comorbid opioid use disorder	☐	☐	☐	☐	☐
g. Patient specifically requested a medication for AUD	☐	☐	☐	☐	☐

h. Patient is participating in a formal treatment program for AUD	☐	☐	☐	☐	☐
i. Patient is willing to comply with a medication to treat AUD	☐	☐	☐	☐	☐
j. Patient has insurance coverage for medication	☐	☐	☐	☐	☐

E. OPINIONS ABOUT MEDICATION ATTRIBUTES

E1. Please rate the effectiveness of the following medications in addressing the main symptoms of the indicated disease.

	Poor	Fair	Good	Very Good	Excellent	Don't Know
a. Oral Naltrexone (ReVia or Depade) for AUD	☐	☐	☐	☐	☐	☐
b. Long-Acting Naltrexone (Vivitrol) for AUD	☐	☐	☐	☐	☐	☐
c. Disulfiram (Antabuse) for AUD	☐	☐	☐	☐	☐	☐
d. Acamprosate (Campral) for AUD	☐	☐	☐	☐	☐	☐
e. Topiramate (Topamax) for AUD	☐	☐	☐	☐	☐	☐
f. Baclofen (Lioresal) for AUD	☐	☐	☐	☐	☐	☐
g. SSRI or SNRI for major depression	☐	☐	☐	☐	☐	☐
h. Buprenorphine (Suboxone) for opioid use disorder	☐	☐	☐	☐	☐	☐

E2. Please rate the severity of side effects for each of the following medications

	Very severe/ Strongly limits prescribing	Severe/ Limits prescribing	Some/ Limits my prescribing	Few effects	None	Don't Know
a. Oral Naltrexone (ReVia or Depade) for AUD	☐	☐	☐	☐	☐	☐
b. Long-Acting Naltrexone (Vivitrol) for AUD	☐	☐	☐	☐	☐	☐
c. Disulfiram (Antabuse) for AUD	☐	☐	☐	☐	☐	☐
d. Acamprosate (Campral) for AUD	☐	☐	☐	☐	☐	☐
e. Topiramate (Topamax) for AUD	☐	☐	☐	☐	☐	☐
f. Baclofen (Lioresal) for AUD	☐	☐	☐	☐	☐	☐
g. SSRI or SNRI for major depression	☐	☐	☐	☐	☐	☐
h. Buprenorphine (Suboxone) for opioid use disorder	☐	☐	☐	☐	☐	☐

E3. Please consider one of your typical patients with AUD. Please indicate whether you would most likely prescribe Oral Naltrexone (ReVia or Depade), Long-Acting Naltrexone (Vivitrol), Disulfiram (Antabuse), Acamprosate (Campral), Topiramate (Topamax), Baclofen (Lioresal), or none of these under each of the following scenarios.

	Naltrexone (ReVia or Depade)	Long-Acting Naltrexone (Vivitrol)	Disulfiram (Antabuse)	Acamprosate (Campral)	Topiramate (Topamax)	Baclofen (Lioresal)	Prescribe None	Don't Know
**a. Which medication (if any) would you be most likely to prescribe as a first-line medication if the goal of treatment was abstinence?**	☐	☐	☐	☐	☐	☐	☐	☐
**b. Which medication (if any) would you be most likely to prescribe as a first-line medication, if the goal of treatment was a reduction in alcohol consumption?**	☐	☐	☐	☐	☐	☐	☐	☐
**c. Which medication (if any) would you prescribe as a second-line medication (after the patient fails to respond to a first-line medication) if the goal of treatment was abstinence?**	☐	☐	☐	☐	☐	☐	☐	☐
**d. Which medication (if any) would you prescribe as a second-line medication (after the patient fails to respond to a first-line medication) if the goal of treatment was a reduction in alcohol consumption?**	☐	☐	☐	☐	☐	☐	☐	☐

F. NEW TREATMENT APPROACHES: PHARMACOGENETICS

F1. Please indicate whether you agree or disagree with the following statements regarding <u>genetic testing.</u>

	Strongly Agree	Somewhat Agree	Neutral	Somewhat Disagree	Strongly Disagree	Don't Know
**a. I feel comfortable ordering a pharmacogenetic test to predict risk of adverse events or the likelihood of a treatment response (e.g., *CYP2C9* and antidepressant therapy).**	☐	☐	☐	☐	☐	☐
**b. I have access to genetics expertise when I have a question related to a patient.**	☐	☐	☐	☐	☐	☐
**c. I feel that my genetics training adequately prepared me to order genetic tests and use the results clinically.**	☐	☐	☐	☐	☐	☐
