# Supplementary material for: Survey of Addiction Specialists’ Use of Medications to Treat Alcohol Use Disorder
Source: Front Psychiatry. 2020 Feb 14;11:47. doi: 10.3389/fpsyt.2020.00047 (PMC7034336; doi:10.3389/fpsyt.2020.00047)
Supplement: Supplementary file 2 [file DataSheet_2.docx]

| **Supplementary Table 1: Respondents' Professional Roles (n=501)** | |  |
| --- | --- | --- |
| Respondent profession | Percent of respondents | |
| Physician (non-trainee) | 81.6 | |
| Resident or fellow | 7.2 | |
| Nurse practitioner or physician assistant | 3.8 | |
| Psychologist | 1.8 | |
| Other | 4.2 | |
| Missing | 0.4 | |

| **Supplementary Table 2: Board Certifications of Respondents (n=495)** | |  |
| --- | --- | --- |
| **Participant Board Certifications** | Percent of respondents |  |
| Addiction Medicine (ABAM/ABPM)^a^ | 29.1 |  |
| Addiction Psychiatry (ABPN)^b^ | 17.8 |  |
| Psychiatry (ABPN)^b^ | 14.9 |  |
| Internal Medicine (ABIM)^c^ | 4.6 |  |
| Family Practice (ABFM)^d^ | 7.5 |  |
| Other | 13.5 |  |
| Not board certified in any specialty | 12.5 |  |
| ^a^American Board of Addiction Medicine/American Board of Preventative Medicine | |  |
| ^b^American Board of Psychiatry and Neurology | |  |
| ^c^American Board of Internal Medicine | |  |
| ^d^American Board of Family Medicine |  |  |

| **Supplementary Table 3: Respondents’ ratings of ability to summarize clinical research findings for each medication (n=398)^a^** | | | | | | | | | |
| --- | --- | --- | --- | --- | --- | --- | --- | --- | --- |
| Medication | Never heard of medication | Poor | Fair | | Good | | Very Good | Excellent |  |
| Oral naltrexone (for AUD) | 0 | 4.0 | 8.0 | 21.3 | | 33.6 | | 33.1 |  |
| Long-acting naltrexone (for AUD) | 0.3 | 4.5 | 8.0 | 18.1 | | 32.7 | | 36.4^b^ |  |
| Disulfiram (for AUD) | 0 | 6.8 | 14.2 | 24.8 | | 26.6 | | 27.6^b^ |  |
| Acamprosate (for AUD) | 1.8 | 6.8 | 17.3 | 21.6 | | 28.9 | | 23.6^b^ |  |
| Topiramate (for AUD) | 0.5 | 12.6 | 23.2 | 28.5 | | 20.5 | | 14.6^b^ |  |
| Baclofen (for AUD) | 2.0 | 21.9 | 29.1 | 24.4 | | 11.6 | | 11.^b^ |  |
| SSRI or SNRI (for MDD) | 0 | 3.5 | 8.3 | 17.3 | | 26.6 | | 44.2^b^ |  |
| Buprenorphine (for OUD) | 0 | 1.5 | 2.8 | 7.1 | | 19.6 | | 69.0^b^ |  |
| ^a^Percentage of respondents who endorsed each of the six possible responses. 21% of respondents did not complete this question. | | | | | | | | | |
| ^b^Based on a χ-squared test, the percentage of respondents having excellent ability in summarizing clinical research findings for other medications is significantly different from the corresponding percentage for oral naltrexone at *p* < 0.001. | | | | | | | | | |

| **Supplementary Table 4: Respondents' ratings of research evidence on medications used in the treatment of AUD (n=375)^a^** | | | | | | |
| --- | --- | --- | --- | --- | --- | --- |
| Medication | Strong evidence against using | Weak evidence against using | Mixed evidence | Weak evidence in favor of using | Strong evidence in favor of using | Don't know |
| Oral naltrexone | 1.0 | 1.3 | 6.3 | 21 | 67.8 | 2.5 |
| Long-acting naltrexone | 0.5 | 0.8 | 4.5 | 17.4 | 73.2^b^ | 3.5 |
| Disulfiram | 3.5 | 4.3 | 21.8 | 32.7 | 34.4 | 3.3 |
| Acamprosate | 1.0 | 3.0 | 20.2 | 41.3 | 29.0^b^ | 5.5 |
| Topiramate | 0.8 | 3.5 | 23.2 | 47.9 | 14.4^b^ | 10.3 |
| Baclofen | 2.3 | 7.1 | 34.3 | 32.2 | 2.0 | 22.2 |
| Gabapentin | 1.8 | 6.6 | 21.7 | 39.9 | 20.7 | 9.3 |
| ^a^Percentage of respondents who endorsed each of the six possible responses. Twenty-one percent of respondents did not complete this question.  ^b^Based on a chi-squared test, the percentage of prescribers stating that there is strong evidence in favor of using this medication differs significantly from the corresponding percentage for oral naltrexone (P < 0.001). | | | | | | |

| **Supplementary Table 5: Prescribers’ ratings of the severity of side effects of medications for AUD and other psychiatric medications (n=354)^a^** | | | | | | |  |  |
| --- | --- | --- | --- | --- | --- | --- | --- | --- |
|  | Very severe/ strongly limits prescribing | Severe/ limits prescribing | Some/ limits my prescribing | Few Effects | None | Don't know | |  |
| Oral naltrexone for AUD | 0.6 | 1.4 | 23.6 | 69.1 | 2.8 | 2.5 | |  |
| Long-acting naltrexone for AUD | 0.3 | 2.8 | 26.1 | 65.0 | 1.7 | 4.2 | | ^b^ |
| Disulfiram for AUD | 12.6 | 26.1 | 34.3 | 20.2 | 2.8 | 3.9 | |  |
| Acamprosate for AUD | 0.3 | 2.0 | 19.5 | 59.0 | 9.6 | 9.6 | |  |
| Topiramate for AUD | 0.6 | 9.3 | 48.0 | 27.1 | 1.4 | 13.6 | |  |
| Baclofen for AUD | 1.4 | 5.4 | 31.4 | 27.7 | 2.8 | 31.4 | |  |
| SSRI or SNRI for major depression | 0.3 | 0.8 | 29.7 | 60.5 | 5.1 | 3.7 | | ^b^ |
| Buprenorphine for opioid use disorder | 0 | 1.1 | 14.9 | 70.2 | 11.8 | 2.0 | | ^b^ |
| ^a^Percentage of prescribers who endorsed each of the 6 possible responses. 23% of prescribers did not complete this question. | | | | | | |  |  |
| ^b^Based on a chi-squared test, the percentage of prescribers stating that the side effects of this medication limit their prescribing is significantly different from the corresponding percentage for oral naltrexone at P < 0.001. | | | | | | |  |  |

| **Supplementary Table 6: Respondents' Rating of How Patient Characteristics Influence Prescribing (n=398)^a^** | | | | |  |
| --- | --- | --- | --- | --- | --- |
|  | In favor of prescribing | |  | Against prescribing | |
|  | Strong | Moderate | No effect | Moderate | Strong |
| Patient is willing to comply with a medication regimen to treat AUD | 77.2 | 17.9 | 4.9 | 0 | 0 |
| Patient specifically requested a medication for AUD | 77.2 | 17.1 | 4.6 | 1.1 | 0 |
| Patient is experiencing craving | 73.2 | 22.2 | 4.3 | 0.3 | 0 |
| Patient has experienced a previous relapse | 64.9 | 23.6 | 10.6 | 0.5 | 0.3 |
| Patient is participating in a formal treatment program for AUD | 57.7 | 24.1 | 17.9 | 0.3 | 0 |
| Patient has comorbid opioid use disorder | 57.3 | 22.8 | 12.8 | 5.7 | 1.4 |
| Patient has a history of alcohol withdrawal | 50.1 | 26.6 | 21.4 | 1.4 | 0.5 |
| Patient has insurance coverage for medication | 49.9 | 25.3 | 24.5 | 0.3 | 0 |
| Patient has comorbid mental illness | 45.3 | 32.2 | 17.9 | 4.6 | 0 |
| Patient has impaired liver functioning | 18.4 | 23.6 | 20.6 | 32.8 | 4.6 |
| ^a^Percentage of prescribers who endorsed each of the 5 possible responses. 20% of respondents did not complete this question. | | | | | |

| **Supplementary Table 7: Respondents’ comfort with pharmacogenetics (n=373)^a^** | | | | | |  |
| --- | --- | --- | --- | --- | --- | --- |
|  | Strongly Agree | Somewhat Agree | Neutral | Somewhat Disagree | Strongly Disagree | Don't know |
| I feel comfortable ordering a pharmacogenetic test to predict risk of adverse events or the likelihood of a treatment response. | 11.0 | 21.4 | 17.9 | 18.4 | 20.9 | 10.4 |
| I have access to genetics expertise when I have a question related to a patient. | 10.2 | 21.9 | 12.3 | 15.2 | 31.0 | 9.4 |
| I feel that my genetics training adequately prepared me to order genetic tests and use the results clinically. | 6.7 | 12.9 | 14.2 | 25.5 | 35.1 | 5.6 |
| ^a^Percentage of respondents who endorsed each of the 5 possible responses. 25% of respondents did not complete this question. | | | | | | |
